# Supplementary material for: Cudraxanthone D Ameliorates Psoriasis-like Skin Inflammation in an Imiquimod-Induced Mouse Model via Inhibiting the Inflammatory Signaling Pathways
Source: Molecules. 2021 Oct 8;26(19):6086. doi: 10.3390/molecules26196086 (PMC8512696; doi:10.3390/molecules26196086)
Supplement: Supplementary file 1 [file molecules-26-06086-s001.zip › rRe_Supplementray Table S1.pdf]

Table S1. Primer sequences and antibodies.

In vitro (Human)		
**Primer**	**Sequence (5'→3')**	**GenBank accession number**
CCL17	F: GTT CGG ACC CCA ACA ACA AG	NM_002987.3
	R: TGG CTC CAG TTC AGA CAA GG	
IL-1β	F: GCT GAT GGC CCT AAA CAG ATG AA	NM_000576.3
	R: TGA AGC CCT TGC TGT AGT GGT G	
IL-6	F: AAA GAG GCA CTG GCA GAA AA	NM_001371096.1
	R: ATC TGA GGT GCC CAT GCT AC	
IL-8	F: GGT GCA GTT TTG CCA AGG AG	NM_000584.4
	R: TGC TTG AAG TTT CAC TGG CAT C	
GAPDH	F: CGA CCA CTT TGT CAA GCT CA	NM_002046.7
	R: AGG GGA GAT TCA GTG TGG TG	

In vivo (mouse)		
**Primer**	**Sequence (5'→3')**	**GenBank accession number**
CXCL1	F: TGT GGG AGG CTG TGT TTG TA	NM_008176.3
	R: ACG AGA CCA GGA GAA ACA GG	
IL-25	F: CAG GGA CTT GAA TCG GGT C	NM_080729.3
	R: TGG TAA AGT GGG ACG GAG T	
IL-17A	F: TTT AAC TCC CTT GGC GCA AAA	NM_010552.3
	R: CTT TCC CTC CGC ATT GAC AC	
IL-6	F: GAC TGA TGC TGG TGA CAA CC	NM_0013140541.1
	R: TCA TTT CCA CGA TTT CCC AGA G	
IL-1β	F: ATA ACC TGC TGG TGT GTG AC	NM_008361.4
	R: AGG TGC TGA TGT ACC AGT TG	
IL-36	F: GCT GTG TTG AGA TGG AGG GC	NM_027163.4
	R: GAC AGA AGT GGA GCC CTC TA	
IL-4	F: TCG GCA TTT TGA ACG AGG TC	NM_021283.2
	R: GAA AAG CCC GAA AGA GTC TC	
CD3	F: GCT CCA GGA TTT CTC GGA AGT C	NM_007648.5
	R: ATG GCT ACT GCT GTC AGG TCC A	
IFN-γ	F: TCA AGT GGC ATA GAT GTG GAA GAA	NM_008337.4
	R: TGG CTC TGC AGG ATT TTC ATG	
Ly6G	F: AAC GAA CGTGAT GAACTC CCA	NM_033478.3
	R: TAG TGA TGG CTC AAG GTC GC	
CD11b	F: ATC AAC AAT GTG ACCGTA TGG	NM_008401.2
	R: CAT CAT GTC CTT GTA CTG CCG C	
TLR7	F: GTG ATG CTG TGT GGT TTG TCT GG	NM_001290755.1
	R: CCT TTG TGT GCT CCT GGA CCT A	
GAPDH	F: TGC TCC TCC CTG TTC CAG A	NM_008084.3
	R: TAC GGC CAA ATC CGT TCA CA	

CCL, Chemokine (C-C motif) ligand; CXCL, C-X-C motif chemokine; IL, Interleukin; CD, Cluster of differentiation; IFN, interferon; TLR, Toll like receptor; GAPDH, Glyeraldehyde-3-phosphate dehydrogenase.

Primary antibody	Size (kDa)	Supplier	Cat#	Dilution	Host
Phospho-STAT1^{Y701}	89, 91	Cell Signaling	9167S	1:1000	Rabbit
STAT1	89, 91	Cell Signaling	9172S	1:1000	Rabbit
Lamin B1	67	Santa Cruz	SC374015	1:500	Mouse
NF-κB p65	65	Santa Cruz	SC8008	1:500	Mouse
Phospho-NF-κB p65	65	Cell Signaling	3033S	1:1000	Rabbit
β-actin (C4)	43	Invitrogen	MA5-15739	1:4000	Mouse
IκBα	35-41	Santa Cruz	SC1643	1:500	Mouse
**Secondary antibody**		**Supplier**	**Cat#**	**Dilution**	**Host**
Anti-rabbit IgG		Cell Signaling	7074S	1:1000	Rabbit
Anti-mouse IgG		Cell Signaling	7076S	1:2000	Mouse
