# Supplementary material for: Cudraxanthone D Ameliorates Psoriasis-like Skin Inflammation in an Imiquimod-Induced Mouse Model via Inhibiting the Inflammatory Signaling Pathways
Source: Molecules. 2021 Oct 8;26(19):6086. doi: 10.3390/molecules26196086 (PMC8512696; doi:10.3390/molecules26196086)
Supplement: Supplementary file 1 [file molecules-26-06086-s001.zip › Supplementary 1.pdf]

Cudraxanthone D ameliorates the psoriasis-like skin inflammation in an imiquimod-induced mouse model via inhibiting the inflammatory signaling pathways

Namkyung Kim [1], Soyoung Lee [2], Jinjoo Kang [1], Young-Ae Choi [1], Yong Hyun Jang [3,*], Gil-Saeng Jeong [4,*], Sang-Hyun Kim [1,*]

[1] Cell & Matrix Research Institute, Department of Pharmacology, School of Medicine, Kyungpook National University, Daegu 41944, Republic of Korea; nortonnklab@gmail.com (N.K.); jinjoo1kang@gmail.com (J.K.); korryy@hanmail.net (Y.-A.C.); shkim72@knu.ac.kr (S.-H.K)

[2] Immunoregulatory Materials Research Center, Korea Research Institute of Bioscience and Biotechnology, Jeongeup 28116, Republic of Korea; sylee@kribb.re.kr (S.L.)

[3] Departments of Dermatology, School of Medicine, Kyungpook National University, Daegu 41944, Republic of Korea; yhjang@knu.ac.kr (Y.H.J)

[4] College of Pharmacy, Chungnam National University, Daejeon 34134, Republic of Korea; gsjeong@cnu.ac.kr (G.-S.J)

*Correspondence: shkim72@knu.ac.kr (S.-H.K.), yhjang@knu.ac.kr (Y.H.J), gsjeong@cnu.ac.kr (G.-S.J)

Supplementary material and method

Cell Viability

To assess cell viability, HaCaT cells were seeded in 96-well plates at 1×10^4 cells/well. Cells were treated with CA or CD for 24 h. 3-[4,5-Dimethylthiazol-2-yl]-2,5-diphenyltetrazolium bromide (MTT) solution (20 μL, 1 mg/mL) was added to each well. These plates were incubated at 37 °C for 4 h. The formazan crystals were dissolved using 100-μL DMSO per well, and the absorbance was measured using a spectrophotometer (Molecular Devices, Sunnyvale, CA) at a wavelength of 570 nm.

Drug Screening

HaCaT cells (2×10^5 cells/well in a 24 well plate) were pretreated with CD (0.01, 0.1, or 1 μM) or Dexa (1 μM) for 1 h, then stimulated with TNF-α/IFN-γ (10 ng/mL) for 6 h. RNA was isolated in according using an RNAiso Plus Kit (Takara Bio Inc., Shiga, Japan). Synthesis of cDNA was done using the RevertAid RT kit (ThermoFisher scientific, Waltham, MA) according to the manufacturer's instructions. Subsequently, the expression of IL-6 and IL-1B was confirmed via qPCR (PCR 2X Master Mix; Cellsafe, Yongin, Republic of Korea). mRNA expression was normalized with glyceraldehyde 3-phosphate dehydrogenase. Quantification analysis was performed using the StepOnePlus PCR system software (ThermoFisher scientific) as per the manufacturer's instructions.

Induction of Psoriasis-like Skin Inflammation in Mice

Mice were treated daily with topical application of IMQ cream (5% Aldara™; DONG-A Pharmaceuticals, Seoul, Republic of Korea) on the dorsal skin. All mice were randomly divided in to seven groups: (1) control, (2) 10 mg/kg CD, (3) IMQ, (4) IMQ + 0.1 mg/kg of CD, (5) IMQ + 1 mg/kg of CD, (6) IMQ + 10 mg/kg of CD, (7) IMQ + 1 mg/kg of Dexa. IMQ was applied topically on dorsal skin, and CD or Dexa was given orally for 7 consecutive days. The back fur of the mice was shaved and kept for 24 h,

and then 62.5 mg of IMQ cream was rubbed onto the back skin. CD (0.1, 1, or 10 mg/kg) or dexamethasone (Dexa, 1 mg/kg) were orally administered by gavage for 7 consecutive days.

Figure S1. Chemical structures and anti-inflammatory effect of CA and CD, and experimental design in IMQ-induced mouse model.

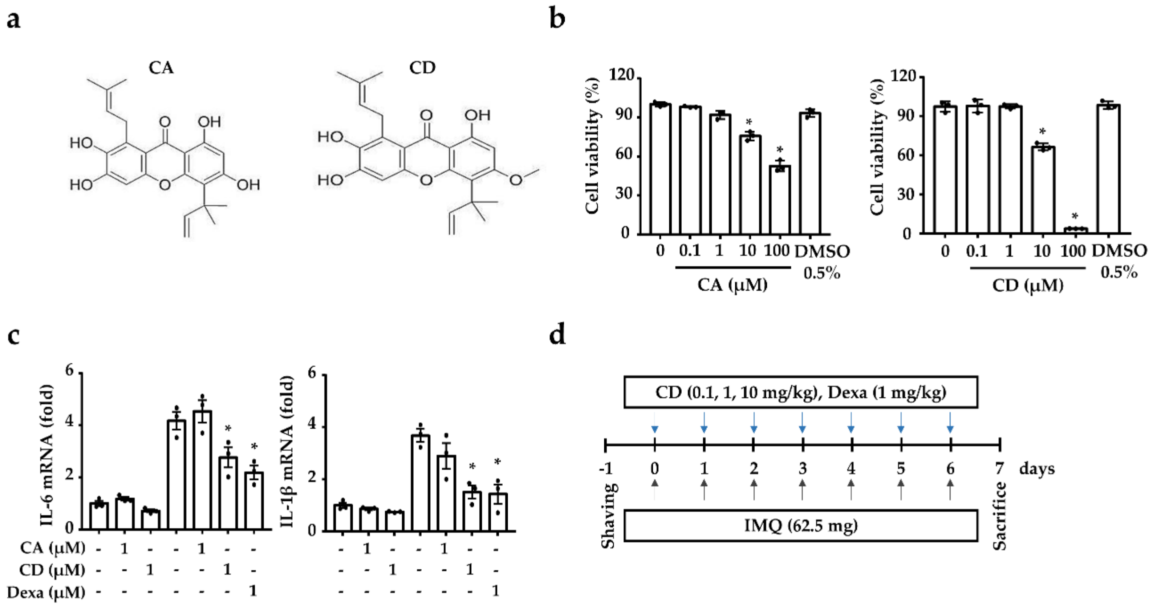

(a) Chemical structures of CA and CD extracted from the root of *C. tricuspidata*. (b) Effects of CA and CD on cell viability. Cell viability was measured via MTT assay. Each data point represents the Mean ± SEM of three independent samples. *$p < 0.05$ compared with the non-treated control group. (c) Gene expression of IL-6 and IL-1β to assess the anti-inflammatory effect of CA and CD in keratinocytes. Each data point represents the Mean ± SEM of three independent samples. *$p < 0.05$ compared with TNF-α/IFN-γ-stimulated group only. (d) The experimental scheme *in vivo*. CA: cudratricusxanthone A, CD: cudraxanthone D, Dexa: dexamethasone, IMQ: imiquimod.
