# Supplementary material for: Cudraxanthone D Ameliorates Psoriasis-like Skin Inflammation in an Imiquimod-Induced Mouse Model via Inhibiting the Inflammatory Signaling Pathways
Source: Molecules. 2021 Oct 8;26(19):6086. doi: 10.3390/molecules26196086 (PMC8512696; doi:10.3390/molecules26196086)
Supplement: Supplementary file 1 [file molecules-26-06086-s001.zip › Supplementary 3.pdf]

Cudraxanthone D ameliorates the psoriasis-like skin inflammation in an imiquimod-induced mouse model via inhibiting the inflammatory signaling pathways

Namkyung Kim [1], Soyoung Lee [2], Jinjoo Kang [1], Young-Ae Choi [1], Yong Hyun Jang [3,*], Gil-Saeng Jeong [4,*], Sang-Hyun Kim [1,*]

[1] Cell & Matrix Research Institute, Department of Pharmacology, School of Medicine, Kyungpook National University, Daegu 41944, Republic of Korea; nortonnklab@gmail.com (N.K.); jinjoo1kang@gmail.com (J.K.); korryy@hanmail.net (Y.-A.C.); shkim72@knu.ac.kr (S.-H.K)

[2] Immunoregulatory Materials Research Center, Korea Research Institute of Bioscience and Biotechnology, Jeongeup 28116, Republic of Korea; sylee@kribb.re.kr (S.L.)

[3] Departments of Dermatology, School of Medicine, Kyungpook National University, Daegu 41944, Republic of Korea; yhjang@knu.ac.kr (Y.H.J)

[4] College of Pharmacy, Chungnam National University, Daejeon 34134, Republic of Korea; gsjeong@cnu.ac.kr (G.-S.J)

*Correspondence: shkim72@knu.ac.kr (S.-H.K.), yhjang@knu.ac.kr (Y.H.J), gsjeong@cnu.ac.kr (G.-S.J)

Supplementary material and method

Protein extraction from IMQ-induced mice skin

At the mice sacrificed, mice skin was gathered in RIPA buffer (Biosesang) containing a protease/phosphatase inhibitor mixture (Roche). Next, mice skin was homogenized by a TissueLyser II (Qiagene). The tissue lysates were centrifuged at 1,200 *g* for 15 min at 4°C, and supernatant was collected. The protein quantification assay was performed with the Bradford Protein assay kit (Bio-Rad Laboratories) and loaded onto 10% sodium dodecyl sulfate polyacrylamide gel electrophoresis, and then transferred onto nitrocellulose membranes (Pall Life science). After transference, membranes were observed with Ponceau S stain. Immunodetection was carried out using SuperSignal West Pico Chemiluminescent Substrate (Thermo Fisher Scientific) by G:BOX Chemi XRQ (Syngene).

Figure S3. Effects of CD on the total and phosphorylated form of STAT1 and NF-κB in mouse skin.

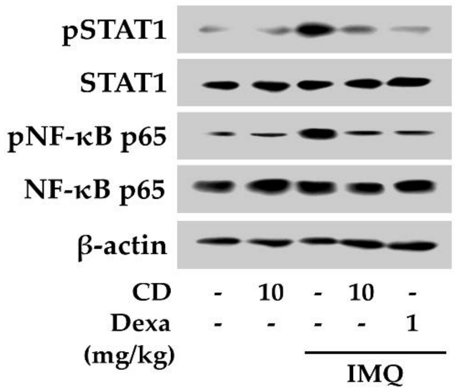

After the *in vivo* experiments, the mice were sacrificed, and their skins were obtained. Activation of STAT1 and NF-κB in IMQ-induced skin was detected via Western blot. The loading control was confirmed using β-actin in independent blots from three randomly-selected skin tissue per each group. CD: Cudraxanthone D, Dexa: dexamethasone.
